# A plant virus (BYDV) promotes trophic facilitation in aphids on wheat

**DOI:** 10.1038/s41598-018-30023-6

**Published:** 2018-08-03

**Authors:** Mitzy Porras, Consuelo M. De Moraes, Mark C. Mescher, Edwin G. Rajotte, Tomás A. Carlo

**Affiliations:** 1 Entomology Department, The Pennsylvania State University, 501 ASI Bldg. University Park, State College, PA 16802 USA; 2 Biology Department, The Pennsylvania State University, 208 Mueller Lab, University Park, State College, PA 16802 USA; 3 Department of Environmental Systems Science, Swiss Federal Institute of Technology (ETH Zürich), CH-8092 Zurich, Switzerland; 4 Intercollege Graduate Ecology Program, The Pennsylvania State University, 208 Mueller Lab, University Park, State College, PA 16802 USA

## Abstract

Pathogens and other parasites can have profound effects on biological communities and ecosystems. Here we explore how two strains of a plant virus – Barley Yellow Dwarf Virus, BYDV – influence the foraging performance and fecundity of two aphid species: *Rhopalosiphum maidis* and *R*. *padi*. We found that pre-inhabitation by *R*. *padi* on plants facilitates the subsequent foraging of conspecifics and *R*. *maidis*. Without the virus, the occurrence of facilitation is asymmetric because it depends on the order of species arrival. However, with virus we found facilitation irrespective of the order of species arrival. Furthermore, the virus also boosted the fecundity of both aphids. Analyses of nutrient content of virus-free and virus-infected plants show significant increases of essential amino acids, sterols, and carbohydrates. Such nutrient increases appear to underlie the facilitative interactions and fecundity of aphids on virus-infected plants. Our experiments demonstrate that the virus dramatically increases the food consumption and fecundity of aphids through intra and interspecific trophic facilitation, resulting in processes that could affect community organization.

## Introduction

Understanding the outcome of interspecific interactions among species with similar ecological requirements is one of ecology’s persisting challenges. Work emerging over the past two decades indicates that parasites can alter the outcome of intra and interspecific interactions^1^. However, our understanding remains limited about the ways in which parasites influence key ecological processes such as community assembly and organization^2,3^. Plant parasites such as viruses might be expected to have large effects on such processes, as plants lie at the center of most community interaction webs and thus mediate direct and indirect interactions among a wide range of organisms^4^. For example, many studies have shown how viruses affect the outcome of competitive interactions^5–7^. However, few studies have examined how viruses affect positive intra and interspecific interactions such as facilitation among phytophagous vector species and its implications for ecological communities^8–10^. Facilitation is any direct or indirect interaction among two or more organisms that increases the fitness of one or more organisms without negatively affecting the other(s)^11^. Phytophagous insect communities associated with grasses provide good experimental systems for the investigation of such questions as they exhibit complex interactions among species that include competition for space and nutrients, that are often mediated by the host plants.

Previous work has established that the order and timing of species colonization can influence the structure and dynamics of communities through facilitation in different types of organisms ranging from bacteria to vertebrates^3,8,10,12–17^. In the case of aphids, for example, small species such as *Rhopaloshipum padi* are first to colonize grasses (e.g., wheat), while others like the larger *R*. *maidis* may follow^18–20^. Studies that have investigated the effects of viruses on the feeding behavior of insect vectors have focused exclusively on intraspecific effects^21,22^ and thus, the consequences of pre-inhabitation and virus infection with respect to interspecific aphid interactions are largely unexplored. For example, foraging activity of a pioneer species like *R*. *padi* might affect the food supply and feeding rates of late-coming species such as *R*. *maidis* in ways that could be negative (competition), neutral, or positive (facilitation).

Here we examine how the order of colonization by two aphid species – *R*. *padi* and *R*. *maidis* – and two virus strains of *Barley yellow dwarf virus* – BYDV-PAV and BYDV-RMV– affect feeding and fecundity. These virus strains are transmitted by aphids in a species-specific manner: *Rhopalosiphum maidis* transmits BYDV-RMV and *R*. *padi* transmits both BYDV-PAV^22,23^. The viruses are transported in the accessory salivary glands of aphids and can be transmitted into plants during feeding for weeks^24,25^. Our null hypothesis was that pre-inhabitation by an aphid species on a host plant and the presence of viruses had no effect on the food consumption and fecundity of a succeeding aphid species. Alternatively, if the chronological order of species arrival and the virus matter, outcomes could range from competition to facilitation, potentially including scenarios in which interactions among species are asymmetric (i.e., only one of the interacting species is harmed or derives benefits from the other). We tested these hypotheses using a combination of field and laboratory experiments that manipulated the presence of virus strains and the order of aphid species arrival to examine (1) the time it takes an aphid to start feeding, (2) the feeding duration, and (3) aphid fecundity. We also examined (4) how the nutritional quality of host plants was affected by aphid foraging and plant virus strains. Our results show that the plant both strains of the virus significantly increase the feeding rates and fecundity of aphids, resulting in niche expansion and changes in the organization of the plant-virus-insect community.

## Materials and Methods

### Insect colonies and Virus

Our laboratory stock colonies of *R*. *padi* and *R*. *maidis* were kept under greenhouse conditions at 20 ± 2 °C and ambient photoperiod. All aphids used in experiments were kept in small colonies (~ten aphids per plant) to avoid stress on individuals associated with high population densities. Spring wheat plants (*Triticum aestivum* L.) were grown from individual seeds planted in a tray of greenhouse cones (4 cm diameter x 21 cm long) with a standard soil substrate with macro- and micro-nutrients (Premier Pro-Mix, Quakertown, PA, USA). Plants were watered twice a day and kept in a growth chamber at 22 ± 1 °C with a 16 h photoperiod (430 µmol m^−2^ s^−1^), 8 h dark period at (20 ± 1 °C), and a relative humidity of 60%. *Virus infection of host plants*. To infect plants, we fed aphids on oat leaves (*Avena strigosa* Schreb) infected with BYDV-PAV and BYDV-RMV during 2–3 days. Then, we exposed healthy wheat plants to the viruliferous aphids for a period of ten days to allow virus inoculation (ten aphids per plant). Infection on each wheat plant was confirmed after 12 days using a double-antibody sandwich, enzyme-linked immunosorbent assay (DAS-ELISA) (Agdia Inc, Elkhart, IN, USA). The virus titer was estimated following the protocol described in Jimenez-Martinez *et al*.^26^ (Table S1).

### Experiment 1: How is aphid foraging behavior affected by pre-inhabitation and virus presence in the host plant?

We used a factorial experimental design to test the hypothesis that pre-inhabitation and viruses affect the foraging behavior of late-coming aphid species. Experimental factors (independent variables) were pre-inhabitation with three levels, and virus infection with three levels. Virus-infection factors cannot be combined between the aphid species because virus transmission is species specific (*R*. *padi* transmits BYDV-PAV and *R*. *maidis* transmits BYDV-RMV). Levels of pre-inhabitation were: pre-inhabited by the same aphid species (intraspecific), pre-inhabited by the other aphid species (interspecific), and control (not pre-inhabited). Levels of virus infection were: host plant infected with BYDV-PAV, host plant infected with BYDV-RMV, intraspecific control (pre-inhabited by the same species but no virus), and interspecific control (pre-inhabited by the other species and no virus). We did not include a double-negative control (i.e., no virus and no pre-inhabitation) because virus inoculation only occurs through aphid foraging. The experimental unit was an individual spring wheat plant (*Triticum aestivum* L.) not previously used in any experiment, and not used again in any other experiment (independent experimental unit). There was a total of six replicates – six different plants – for each unique combination of pre-inhabitation and possible virus-aphid treatments.

To create aphid pre-inhabitation treatments without viruses, we did the following. For the intraspecific pre-inhabitation treatment, we transferred ten 5-day old adults of the same aphid species onto a healthy 3-week old wheat plant and allowed them to feed for 12 days before being removed. Offspring were removed daily to control for the effect of population density. For the interspecific pre-inhabitation treatment, we exposed wheat plants to ten 5-day old adults from the other aphid species for 12 days.

To create the treatments of plants infected with BYDV-PAV or BYDV-RMV viruses, we did the following. For the intraspecific virus-infected treatments, we transferred ten viruliferous aphids onto each experimental wheat plant (healthy 3-week old) and allowed them to feed for a period of 12 days removing their offspring daily. We chose this duration because a successful virus-inoculation can require aphids feeding on the plant up to 10–12 days. For the interspecific pre-inhabitation treatments, we did the same, but with viruliferous aphids of the other species.

We measured two key parameters of aphid foraging as response variables in all treatments: the time taken by aphids to reach the wheat plant’s phloem, and the duration of ingestions. Both responses were measured using electrical potential graphing (EPG). In each trial (i.e., independent replicate wheat plant, *N* = 6 for all treatment combinations) we placed a single adult aphid connected to a channel of a Giga-8 Direct Current-EPG (EPG-Systems, Wageningen, The Netherlands) for a period of eight hours. The Giga-8 DC-EPG system has two electronic components, a voltage source and an input resistor connected to each other. The substrate electrode (output wire) is placed in the soil where the plant is rooted. The other electrode (gold wire 3.0 cm long, 8 µm in diameter) is attached to the insect’s dorsum with a small drop of silver glue – a conductive adhesive^27^. The circuit is completed when the insect’s mouth parts enter the plant and the current flows from the voltage source to the plant and through the insect, the input resistor, and back to the voltage source. The voltage changes resulting from this “biological resistance” are monitored by a computer that graphs and records the values of the insect-plant resistor. Wave forms generated by voltage changes are associated with biological processes involved in feeding behavior such as plant cuticular surface probing, salivation, time to reach the phloem, duration of ingestion, among others^28^. Our EPG recordings started one hour after the aphid was placed on a horizontally oriented leaf, and continued for eight hours. We analyzed the electrical waves associated to each behavior using software (*Stylet* +*a* & d version 1.2) developed by EPG Systems (Wageningen, Netherlands). For more details on virus inoculation see Supplementary section 1: Supplementary Table 1.

### Experiment 2: Is the fecundity of aphid populations affected by pre-inhabitation and the virus?

Here we also used a factorial design in two experiments – one in the laboratory and one in field conditions – to test the hypothesis that pre-inhabitation and viruses affect aphid species’ fecundity for any late-coming aphid species. Experimental factors (independent variables) were exactly as in experiment 1 for both the laboratory and field experiments. However, this time to create pre-inhabitation treatments we exposed each 3-week old wheat plant to 10 adult aphids (virus-free or viruliferous depending on treatment) for 10 days before removing them (the offspring were removed every day). Next, both in the lab and the field, we placed a 1^st^ instar aphid nymph on every replicate host plant per treatment before immediately caging each plant individually with a transparent acrylic tube (4.5 cm diameter × 35 cm length) having two windows (3 cm × 5 cm) made of Lumite fabric (OHCO, Georgia, GA, USA). Each combination of pre-inhabitation and virus treatments had 15 replicate wheat plants in both the lab and the field experiment. As a response variable in both experiments we recorded the total number of offspring produced for 30 days.

### Experiment 3: Do aphid foraging and the plant virus affect the nutritional quality of host plants?

To test the hypothesis that aphid inhabitation and virus infection increase the nutritional quality of host plants, we modified the same general design used in experiments 1 & 2 in order to compare the nutritional content of wheat plants never exposed to aphids or viruses (controls) with plants infected with viruses and inhabited with our aphid species (treatments). To perform this experiment, we grew 360 wheat plants in four trays in the laboratory at a density of 90 plants/tray. From these trays, we assigned 32 randomly chosen plants from across all trays to each unique experimental factor combination of aphid inhabitation and virus presence (total individual plants = 5 aphid-virus treatments x 32 plants per treatment = 160). Note that in this experiment the aphid treatments of interest are not “pre-inhabited” but just “inhabited” by *R*. *padi* or *R*. *maidis*, as there were no aphid foraging treatments following pre-inhabitation by the same or the other species of aphid.

Each plant (experimental unit) was caged in the aforementioned way, and exposed to 10 adult aphids for ten days according to species (*R*. *padi*, *R*. *maidis*, negative control) and virus treatments (BYDV-PAV, BYDV-RMV, negative control). After 10 days of exposure to treatments, we combined all the leaves from eight wheat plants from each treatment to produce one sample for nutrient analysis. Leaves were preserved in liquid nitrogen at −80 °C immediately after harvesting. Thus, although there were 32 independent experimental plants from each treatment, the final replication per treatment was four because of the biomass requirement of chemical analyses. In each sample, we measured the concentration of simple sugars (sucrose and glucose), total sterol content, and amino acids (alanine, arginine, aspartic acid, asparagine, glutamine, glycine, isoleucine, leucine, lysine, methionine, phenylalanine, serine, threonine, tryptophan, tyrosine, and valine) using gas chromatography mass spectrometry or liquid chromatography mass spectrometry analysis (Supplementary section 1).

## Data Analysis

The data from the experiments were analyzed using a Multivariate Analysis of Variance (MANOVA) and Wilks’ lambda. Significant differences in MANOVA were followed by individual analyses of the response parameters (i.e., feeding behavior, fecundity, and nutritional host plant quality) per aphid species, by pre-inhabitation type (intra and interspecific), and by type of virus present^29^. For the experiment 1, we used post-hoc paired *t-*tests to determine whether differences in foraging parameters (e.g., time taken by the aphids to reach the phloem, and duration of ingestion) of the treatments (pre-inhabited or pre-inhabited with virus) against their respective controls (not pre-inhabited, pre-inhabited but without virus) were significant.

For the data from experiment 2, we compared the fecundities among treatments using one-way ANOVAs for each aphid species in each location (field, laboratory), followed by post-hoc Tukey-Kramer HSD mean comparisons.

For experiment 3 we used a completely randomized design to compare the effect of viruses on the nutritional quality of wheat plants. Differences in sugars, total sterols, and amino acids were tested using one-way ANOVA followed by Dunnett’s test comparing treatments against the control plants (i.e., without aphid foraging and virus). All analyses were performed in Python programming language (version 3.5.2). The data that support the findings of this study are available on AEKOS data repository (Data Cite DOI 10.4227/05/5a555d6cc6165).

## Results

### Experiment 1: How is aphid foraging behavior affected by pre-inhabitation and virus presence in the host plant

**Figure 1:**
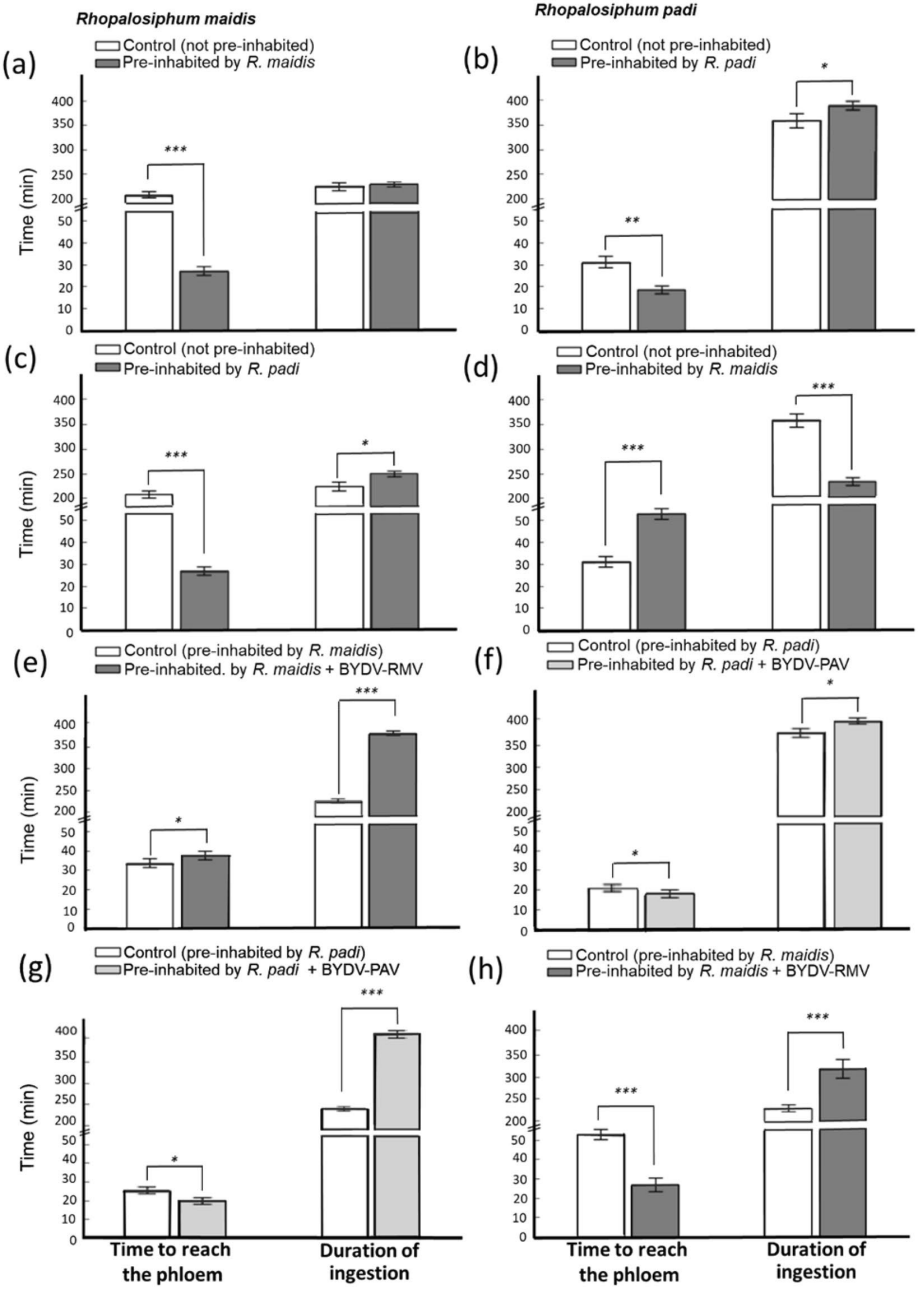
Effects of intra and inter-specific pre-inhabitation of host plants on aphid foraging behavior. Pre-inhabitation by *R*. *padi* reduced the time to reach the phloem and increased the time of ingestions of *R*. *maidis* on wheat plants, but pre-inhabitation by *R*. *maidis* significantly reduced foraging performance for *R*. *padi* showing asymmetric species facilitation. White bars are controls and grey bars are pre-inhabited host plants. (**a**) R. *maidis* feeding behavior in a pre-inhabited host by *R*. *maidis*. (**b**) R. *padi* feeding behavior in a pre-inhabited host by *R*. *padi*. (**c**) *R*. *maidis* feeding behavior in a pre-inhabited host by *R*. *pad*, (**d**) *R*. *padi* feeding behavior in a pre-inhabited host by *R*. *maidis*. *Effects of pre-inhabitation of viruliferous aphids and virus presence*. Note that viruses transmitted by early-arriving species, *R*. *padi*, modify feeding behavior of conspecifics and *R*. *maidis*, increasing ingestion times. The *x*-axis shows control (pre-inhabited hosts virus-free), and plants pre-inhabited by viruliferous *R*. *maidis* carrying BYDV-RMV, *R*. *padi* carrying BYDV-PAV. (**e**) *R*. *maidis* feeding behavior on plants pre-inhabited by *R*. *maidis* with BYDV-RMV, (**f**) *R*. *padi* feeding behavior on plants pre-inhabited by *R*. *padi* with BYDV-PAV, (**g**) *R*. *maidis* feeding behavior on plants pre-inhabited by *R*. *padi* and BYDV-PAV, (**h**) *R*. *padi* feeding behavior on plants pre-inhabited by *R*. *maidis* with BYDV-RMV. White bars for pre-inhabitation without virus, and gray bars for pre-inhabitation of viruliferous aphids. Bars represent means ± SE (*N* = 6). Significance was determined with *t*-tests (* = differences at α = 0.05–0.01; ** = differences at α = 0.01–0.001; *** = differences at α ≤ 0.0001).

Our final dataset consisted of 480 h of feeding behavior records. For both aphid species, feeding behavior parameters significantly differed between pre-inhabited and control plants (MANOVA: Wilks’ Lambda = 0.0019, *F*_18,98_ = 117.26, *P* ≤ 0.0001). *Rhopalosiphum maidis* reached the phloem significantly faster on plants previously foraged by conspecifics (*t* = 24.56, df = 10, *P* ≤ 0.0001), but we did not detect any significant effect on the duration of ingestion (*t* = 1.46, df = 10, *P* = 0.1841; Fig. 1a). The same trend was also observed for *R*. *padi* (*t* = 3.57, df = 10, *P* = 0.0051; *t* = 0.75, df = 10, *P* = 0.4651; Fig. 1b). Pre-inhabitation of host plants by *R*. *padi* significantly decreased the time taken by *R*. *maidis* to reach the phloem by more than three-fold when compared to control plants (*t* = 24.84, df = 10, *P* ≤ 0.0001; Fig. 1c). Furthermore, pre-inhabitation by *R*. *padi* increased the duration of *R*. *maidis* ingestion by 17% (*t* = 3.22, df = 10, *P* = 0.0091; Fig. 1c). Pre-inhabitation by *R*. *maidis* increased the time taken by *R*. *padi* to find the phloem (*t* = 10.18, df = 10, *P* ≤ 0.0001; Fig. 1d), while the duration of ingestion was reduced by 34% (*t* = 8.50, df = 10, *P* ≤ 0.0001; Fig. 1d).

We found that the presence of virus strains altered the feeding behavior of both aphid species, pre-inhabitation of *R*. *maidis* on plants infected with BYDV-RMV increased the duration of ingestion by 37% on conspecifics (*t* = 19.57, df = 10, *P* ≤ 0.0001; Fig. 1e). Pre-inhabitation by viruliferous *R*. *padi* and BYDV-PAV infection reduced the time taken by conspecifics to reach the phloem by 13% (*t* = 40.87, df = 10, *P* = 0.0099; Fig. 1f), and increased the ingestion time by 10% (*F*_2,15_ = 6.28, *P* = 0.0104; Fig. 1f). Also, pre-inhabitation by viruliferous *R*. *padi* with BYDV-PAV reduced the time taken by *R*. *maidis* to reach the phloem by 27% (*t* = 3.13, df = 10, *P* = 0.0105) and increased the time of ingestion by 57% (*t* = 13.13, df = 10, *P* < 0.0001; Fig. 1g). Following a similar trend, the pre-inhabitation by viruliferous *R*. *maidis* with BYDV-RMV significantly decreased the time taken by *R*. *padi* to reach the phloem by 56% (*t* = 8.75, df = 10, *P* < 0.0001; Fig. 1h) and increased the duration of ingestion by 27% (*t* = 3.98, df = 10, *P* = 0.0026; Fig. 1h).

### Experiment 2: Is the fecundity of aphid populations affected by pre-inhabitation and viruses?

**Figure 2:**
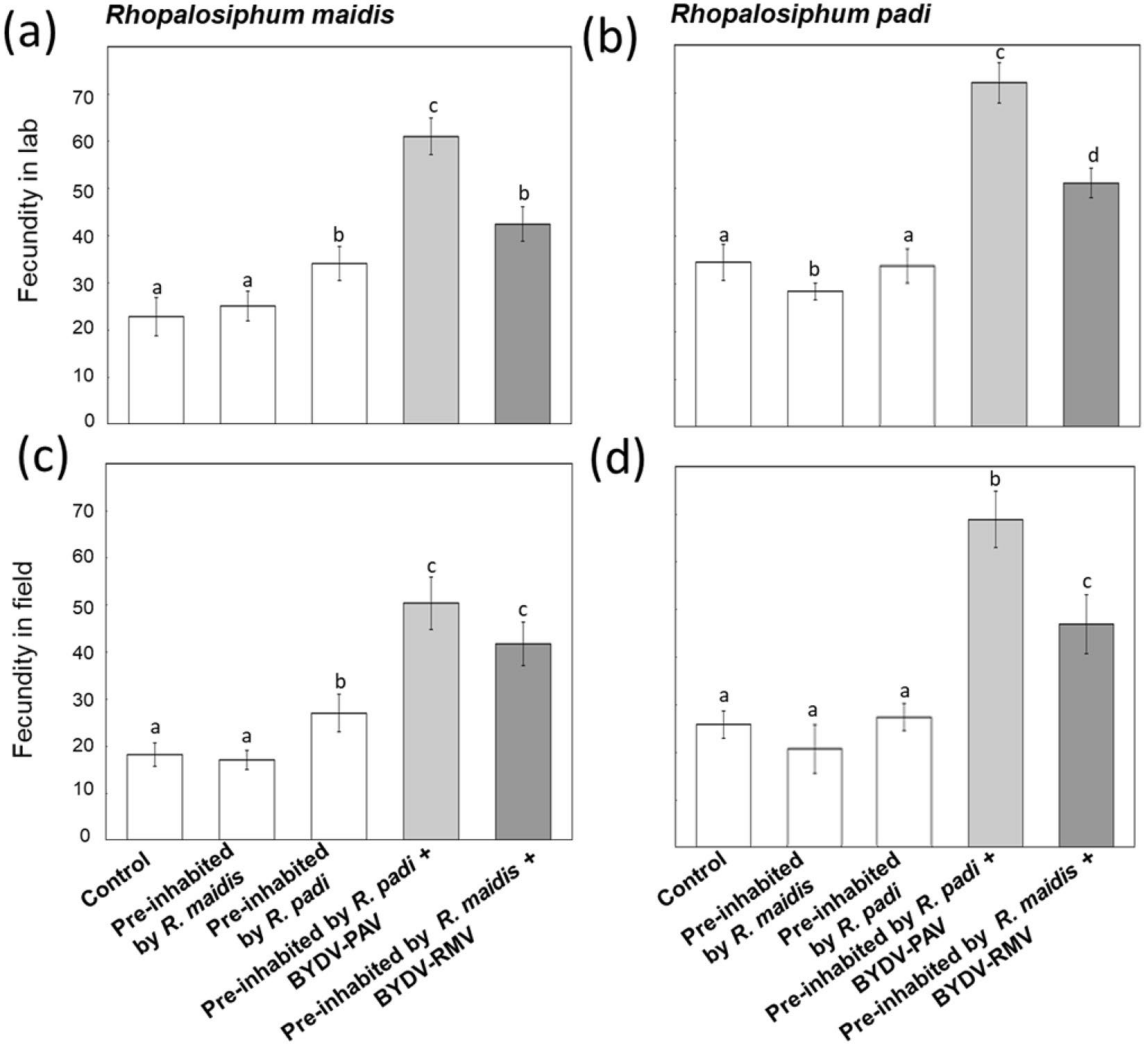
Testing the effects of pre-inhabitation and viruses on fecundity of two aphid species. Pre-inhabitation with viruses had a positive effect on fecundity of both aphid species in laboratory and field conditions. Shown are the mean (±SE) fecundities of each aphid species on pre-inhabitation virus-free and pre-inhabitation with viruses against control (plants without pre-inhabitation). Fecundity in laboratory: (**a**) *R*. *maidis*: *F*_4,70_ = 336.18, *P* < 0.0001; (**b**) *R*. *padi*: *F*_4,70_ = 425.73, *P* < 0.0001. Fecundity in field: (**c**) *R*. *maidis*: *F*_4,70_ = 192.28, *P* < 0.0001; (**d**) *R*. *padi*: *F*_4,70_ = 237.63, *P* < 0.0001. Significance was determined with one-way ANOVAs, followed by Tukey’s HSD tests (*P* < 0.05) (*N* = 15).

Populations of both *R*. *padi* and *R*. *maidis* were able to colonize and reproduce on virus-free plants that were pre-inhabited (MANOVA: Wilks’ Lambda = 0.0017, *F*_16,205_._33_ = 89.08, *P* ≤ 0.0001; Fig. 2). When wheat plants were pre-inhabited by *R*. *padi* carrying BYDV-PAV, *R*. *padi* fecundity increased by 150% and by 125% in *R*. *maidis* relative to controls (Fig. 2a and d, Supplementary information 2: Supplementary Table 2). In contrast, the average fecundity of *R*. *padi* decreased 25% in plants pre-inhabited by *R*. *maidis* (Fig. 2d). However, fecundity of both aphid species increased in plants pre-inhabited by *R*. *maidis* and infected with BYDV-RMV strain. The same trend was also observed in the field (Fig. 2c,d).

### Experiment 3: Do aphid foraging and plant viruses affect the nutritional quality of host plants?

**Figure 3:**
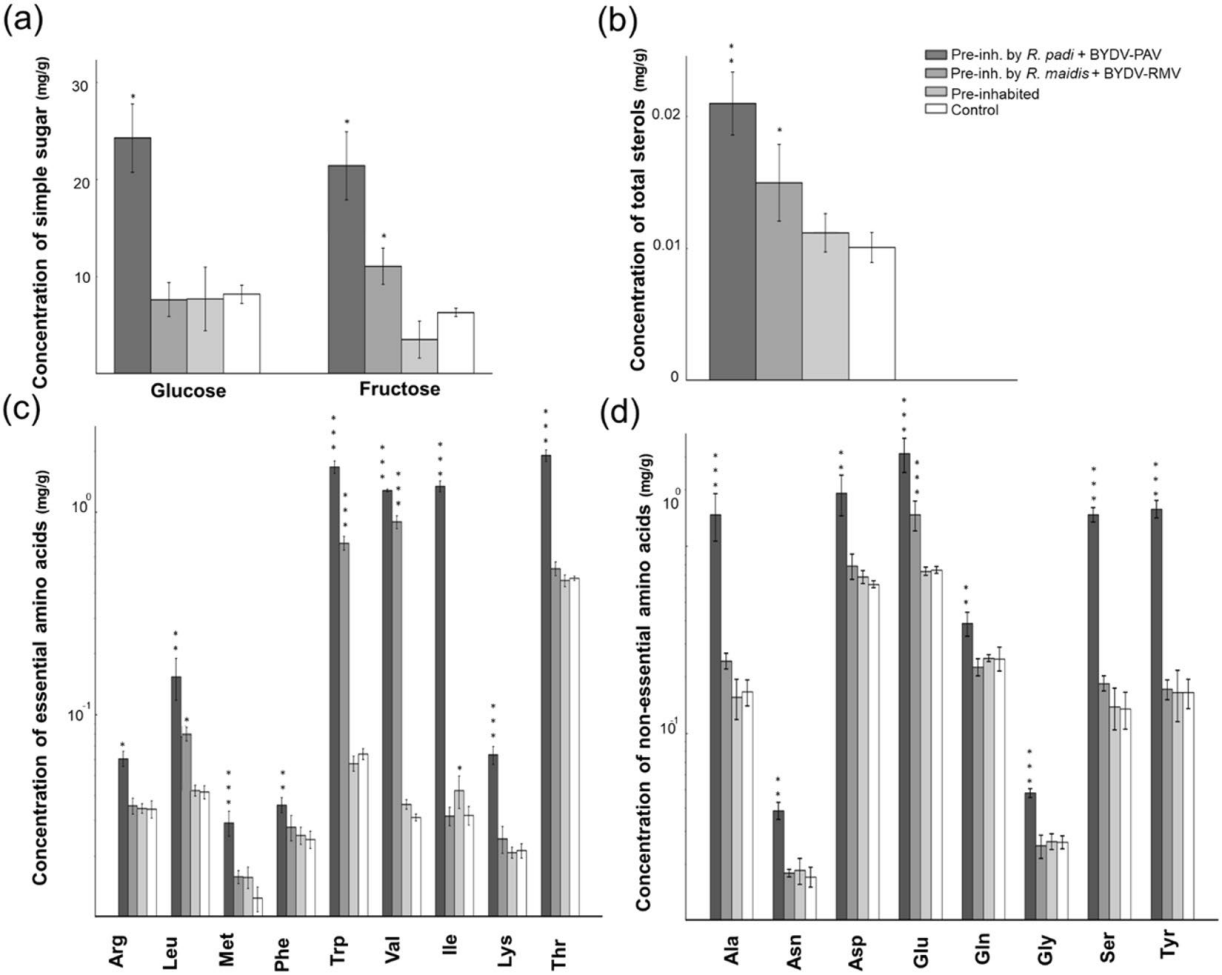
Viruses transmitted by *R*. *maidis* and *R*. *padi* increased the availability of nutrients in wheat leaves relative to control plants consisting of uninhabited virus-free plants (Dunnett’s test comparing each nutrient type to control group, * = differences at α = 0.05–0.01; ** = differences at α = 0.01–0.001; *** = differences at α ≤ 0.0001). (**a**) Simple sugars, (**b**) total sterols, and amino acids: (**c**) essential, (**d**) non-essential for aphids (*N* = 4).

The presence of viruses increased the nutritional content of wheat leaves, specifically the free carbohydrates, sterols, and amino acids (Fig. 3, Supplementary information 2: Supplementary Table 3). The concentration of fructose and glucose increased three-fold in plants with BYDV-PAV, and about two-fold in plants with BYDV-RMV (Fig. 3a). Concentrations of total sterols also increased in pre-inhabited wheat plants infected with BYDV-PAV strain (Fig. 3b). Similarly, we observed large increases in amino acid concentration in pre-inhabited plants with BYDV-PAV in non-essential (asparagine, aspartic acid, glutamic acid, serine, tyrosine) and essential amino acids (leucine, methionine, phenylalanine, tryptophan, isoleucine, lysine, and threonine (Fig. 3c,d).

### Discussion

Our experiments demonstrate that pre-inhabitation of a host plant by an aphid species can produce both intra and interspecific facilitation. Results also show that plant viruses magnify the facilitative effects that plant pre-inhabitation has on late-coming species.

Particularly, the virus strain transmitted by *R*. *padi* – the typical pioneer species – significantly increased the foraging capacity and fecundity of late coming *R*. *maidis* aphids. Furthermore, plant virus had a strong effect on the symmetry of the interspecific interactions. For example, without the virus, interspecific facilitation is asymmetric because pre-inhabitation by *R*. *maidis* hampers the foraging performance of *R*. *padi*. However, *R*. *padi* had no problem inhabiting areas previously occupied by *R*. *maidis* when the BYDV-RMV strain – exclusively transmitted by *R*. *maidis* – was present in plants. Although facilitation is a well-recognized process influencing the assembly of bacteria^12^, protozoan^30^, fungal^15,31,32^, plant^13,29,32^, animal^33^ and mutualistic communities^34^, this is the first time that it is shown to be mediated by a virus in a community of putative insect competitors.

Virus strains changed host plants in ways that increased the fitness of both aphid species. Aphids fed for longer periods and boosted their fecundity in the presence of virus strains (Fig. 2). But virus strains changed more than just the feeding behavior of aphids, they also created simultaneous changes in the nutrient environment of host plants, especially the virus strain transmitted by the pioneer aphid *R*. *padi* (Fig. 3). For example, compared to controls, the average concentration of sugars was at least twice as high in plants infected with BYDV-PAV. Additionally, pre-inhabitation and virus infection may induce changes in the leaf anatomy^35,36^ possibly shortening the distance between the vascular bundles and the surface of the leaf that serve as a foraging cue, facilitating foraging for later arriving aphids^37^.

Underscored are the significant increases of nutrients like plant sterols in virus-infected plants (Fig. 3). Sterols are important nutrients for all insects because they are critical component of cell membranes and the required precursors for insect molting hormone, and insects cannot synthesize them^38,39^. Higher concentrations of sterols are also correlated to enhanced fitness in aphids^34,40^. In addition to sterols and sugars, we found significant increases in the concentration of amino acids in our virus-infected plants (Fig. 3). Indeed, at least two previous studies also reported increases in sugars and amino acids in wheat and barley plants infected with the BYDV-PAV virus^35,41^ However, the concentrations reported in the present study are lower than the previously reported, possibly due to differences in growth conditions, strain virulence, or method of detection. Still, our analysis of the nutritional content of plant leaf tissues are only a proxy for the potential effects of viruses on plant physiology as it relates to phloem-feeding insects since ideally the phloem sap should be collected for plant nutritional content analysis^42,43^. Thus, since aphids are specialist phloem-feeders, additional studies are needed to study in more detail how changes in sap nutritional content affects aphid interactions and population dynamics.

For both aphid species, viruses increased the magnitude and symmetry of interspecific facilitative effects of prior foraging, although some outcomes were species-specific. For example, in the absence of virus, prior foraging by *R*. *maidis* had a negative impact on the foraging behavior and fecundity of a late-coming *R*. *padi* (Figs 1 and 2). But the BYDV-RMV virus changed this effect to a positive one, increasing fecundity for the late-coming *R*. *padi* to the same levels reached on control plants in the field, or attaining higher fecundity than controls in laboratory conditions (Fig. 2). In contrast, viruses transmitted by *R*. *padi* had consistently larger positive effects on the fecundity of any succeeding aphid species compared with the virus transmitted by *R*. *maidis*.

Our results suggest that viruses can play an important role in changing the nature of intra and interspecific interactions of competing species by promoting trophic facilitation, and affecting processes of community assembly. The longer feeding times aphids exhibit when exposed to a pre-inhabited virus-infected host should increase probabilities of virus acquisition and transmission^44,45^. Thus, our results add to the mounting evidence that viruses and other pathogens modify the behavior of animal vectors in ways that are conducive to fitness gains for the pathogen. More research is necessary to further uncover the implications of this type of animal-plant-pathogen interactions on the stability and coexistence of insect populations, and the dynamics of diseases given the improbable situation where, for competing insects – “the enemies of our food made us friends”.

## Electronic supplementary material

Supplementary information 1: Methods

Supplementary information 2 Tables
