# Supplementary material for: A plant virus (BYDV) promotes trophic facilitation in aphids on wheat
Source: Sci Rep. 2018 Aug 3;8:11709. doi: 10.1038/s41598-018-30023-6 (PMC6076312; doi:10.1038/s41598-018-30023-6)
Supplement: Supplementary file 1 — Supplementary information 1: Methods [file 41598_2018_30023_MOESM1_ESM.docx]

A plant virus (BYDV) promotes trophic facilitation in aphids on wheat

Supplementary information 1: Methods

Mitzy Porras^1,2*^, Consuelo De Moraes ^2,3^, Mark Mescher ^2,3^, Edwin Rajotte^1^, Tomás Carlo^2, 4^

^1^ Entomology Department, The Pennsylvania State University, 501 ASI Bldg. University Park, PA 16802, USA

^2^ Biology Department, The Pennsylvania State University, 208 Mueller Lab, University Park, PA 16802, USA

^3^ Department of Environmental Systems Science, Swiss Federal Institute of Technology (ETH Zürich), CH-8092 Zurich, Switzerland

^4^Intercollege Graduate Ecology Program, The Pennsylvania State University, 208 Mueller Lab, University Park, PA 16802, USA

*Corresponding author: Mitzy Porras, E-mail: [mfp145@psu.edu](mailto:mfp145@psu.edu)

**Table S1** Virus concentration of wheat leaves of transmitted by *R. maidis* and *R. padi* of infected plants used in experiments 1, 2 and 3. Shown are the mean (± SE) for each virus concentration (ng).

| **Virus species** | **Mean** | **SE** |
| --- | --- | --- |
| **Experiment 1: How is aphid foraging behavior affected by pre-inhabitation and virus presence in the host plant?** | | |
| BYDV-PAV | 502.42 | 33.88 |
| BYDV-RMV (Bt) | 262.97 | 25.42 |
| **Experiment 2: Is the fecundity of aphid populations affected by pre-inhabitation and the virus?** | | |
| BYDV-PAV | 687.17 | 20.45 |
| BYDV-RMV (Bt) | 203.16 | 18.30 |
| **Experiment 3: Do aphid foraging and the plant virus affect the nutritional quality of host plants?** | | |
| BYDV-PAV | 426.51 | 8.96 |
| BYDV-RMV (Bt) | 199.44 | 20.05 |

**Protocols to assess the effect of pre-inhabitation and virus infection on nutritional condition of wheat plants**: We measured carbohydrates (sucrose and glucose), sterols (campesterol, stigmasterol, sitosterol), and amino acids (alanine, arginine, aspartic acid, asparagine, glutamine, glycine, isoleucine, leucine, lysine, methionine, phenylalanine, serine, threonine, tryptophan, tyrosine, and valine) using the following protocols: *Carbohydrate analysis*, we performed a protocol modified from Campbell *et al*. ^1^ ~100 mg plant tissue placed in a 2 mL tube with 650 μL methanol and 750 mLwater, then homogenized in a tissue lyser at 20 Hz for 20 min with a 4 mm stainless steel ball. We added 5000 mL chloroform and vortexed for 15 min and centrifuged at maximal speed for three minutes. The aqueous phase was removed and sample dried. Then, we added 100 mL TriSil and incubated the samples at 70 ˚C for ten minutes with shaking, and diluted tenfold in hexane, spin to remove particles, and loaded into gas chromatography vials. The samples were analyzed by gas chromatography mass spectroscopy (GC-MS) with a column RX 5 msi (30 m x 250 μm x 0.25 μm; Phenomenex, Torrence, CA, USA), with equilibration time 0.2 min. The oven program was 168 ˚C for 8.9 min, then 30 ˚C min^-1^ to 255 ˚C, 1 ˚C min^-1^ to 270 ˚C, 100 ˚C to 300 ˚C for 1 min, then 120 ˚C min^-1^ to 168 ˚C for 1 min; run time 30.2 min, 0.2 min (post run) at 168 ˚C, flow rate (He) 1 mL min^-1^. Front SS inlet He: mode splitless, heather on 250 ˚C. MS data acquired in scan mode 40 - 1000.

*Sterols analysis*, we performed a protocol as described by Phillips *et al*.^2^ ~100 mg plant tissue was placed in a two ml tube with 375 μL MEoH hexane/ 2 μM cholesterol (Sigma-Aldrich, St Louise, MO, USA) and 750 μL water. Then we homogenized in the tissue in a lyser at 20 Hz for 20 min with a 4 mm stainless steel ball, and centrifuged at maximal speed for three minutes. To separate organic and aqueous phases, we dried the organic phase and reconstituted in 75 μL hexane for GC-MS. This fraction contained free sterols. Then, we dried the aqueous phase and reconstitute in 375 μL 90% MeOH/10% HCl/ 2 μM cholesterol; shook at 1400 rpm at 55 ˚C on the thermomixers for 2.5 h. This hydrolyzes glycosylated sterols to free sterols, then we added internal standards (Sigma-Aldrich St Louise, MO, and Avanti Polar Lipids, Inc., Alabaster, Alabama, AL, USA). We dried the hydrolyzed fraction and reconstitute in 75 μL hexane for GC-MS analysis. The instrument had a column RX 5 msi (30 m x 250 µm x 0.25 µm; Phenomenex, Torrence, CA, USA), we used an equilibration time of 0.2 min, and the following method: 200 °C for 0.2 min, then 20 °C min^-1^ to 330 °C for 7 min, 110 °C min^-1^ to 200 °C for 0.2 min, run time 15.08 min, post run time 0.2 min at 200 °C, flow rate (He) 1 mL min^-1^, injection volume 1 µL. Front SS Inlet He mode Splitless, heater at 250 °C, Thermal Aux 2 {MSD Transfer line} 300 ˚C, and MS data acquired in scan mode 35-400. A*mino acid analysis.* We measured amino acids as described by Hacham *et al*.^3^ placed the samples in a reaction vessel containing 1 mL of 6N HCl. The vessel was purged with nitrogen. The samples were incubated for 24 h at 110 ˚C. Upon hydrolysis, we freeze-dried the samples again. The resulting amino acids were reconstituted in 0.4 mL 0.1N HCl. Derivatization: 10 μL of extract and 2 μL of uniformly labelled amino acid standard (Cambridge Isotope Labs part no CDNLM-6784) solution were added to 0.2 mL 0.2M borate buffer (pH 9). 20 μL of reconstituted AccQ-Tag reagent was added to each vial. Solutions were incubated for 10 min at 50 ˚C. Liquid chromatography mass spectrometry (LC-MS/MS) analysis: we used Water/micromass Quatro LC mass spectrometer interfaced to an Agilent 1100 fitter with Waters AccQ-Tag 3.9 x 150 mm column. The mobile phase A was 10 mM ammonium formate in 0.1% formic acid and mobile phase B was acetonitrile. Flow rate was constant at 1 m min^-1^. The gradient separation was time (min) –B: 0-5, 2-5, 15-30, 20-60, 21-60, 22-5, and 30-5.

**References**

1. Campbell, J.A., Donald, P. & Goheen, S.C. Extraction and analysis of inositols and other carbohydrates from soybean plant tissues. INTECH Open Access Publisher. (2011).

2. Phillips, K.M., Ruggio, D.M. & Bailey, J.A. Precise quantitative determination of phytosterols, stanols, and cholesterol metabolites in human serum by capillary gas–liquid chromatography. *J Chromatogr B Biomed Sci Appl.* **732,** 17–29 (1999).

3. Hacham, Y., Avraham, T. & Amir, R. The N-terminal region of Arabidopsis cystathionine γ-synthase plays an important regulatory role in methionine metabolism. *Plant Physiol.* **128,** 454–462 (2002).
