# Supplementary material for: A plant virus (BYDV) promotes trophic facilitation in aphids on wheat
Source: Sci Rep. 2018 Aug 3;8:11709. doi: 10.1038/s41598-018-30023-6 (PMC6076312; doi:10.1038/s41598-018-30023-6)
Supplement: Supplementary file 2 — Supplementary information 2 Tables [file 41598_2018_30023_MOESM2_ESM.docx]

A plant virus (BYDV) promotes trophic facilitation in aphids on wheat

Supplementary information 2

Tables

Mitzy Porras^1,2*^, Consuelo De Moraes ^2,3^, Mark Mescher ^2,3^, Edwin Rajotte^1^, Tomás Carlo^2, 4^

^1^ Entomology Department, The Pennsylvania State University, 501 ASI Bldg. University Park, PA 16802, USA

^2^ Biology Department, The Pennsylvania State University, 208 Mueller Lab, University Park, PA 16802, USA

^3^ Department of Environmental Systems Science, Swiss Federal Institute of Technology (ETH Zürich), CH-8092 Zurich, Switzerland

^4^Intercollege Graduate Ecology Program, The Pennsylvania State University, 208 Mueller Lab, University Park, PA 16802, USA

*Corresponding author: Mitzy Porras, E-mail: [mfp145@psu.edu](mailto:mfp145@psu.edu)

**Table S2** Effects of pre-inhabitation and virus presence on aphid fecundity. Fecundity of *Rhopalosiphum maidis* and *R. padi*. Dunnet’s test comparing fecundity of each aphid species on each treatment against the control.

| **Treatment** | **Laboratory** | |  | **Field** | |
| --- | --- | --- | --- | --- | --- |
|  | **Absolute difference LSD** | ***P*** |  | **Absolute difference LSD** | ***P*** |
|  | ***Rhopalosiphum maidis*** | | | | |
| *R. maidis* on pre-inhabited host by viruliferous *R. padi* with BYDV-PAV | 35.22 | < 0.0001 |  | 28.39 | < 0.0001 |
| *R. maidis* on pre-inhabited host by viruliferous *R. maidis* BYDV-RMV | 16.62 | < 0.0001 |  | 19.73 | < 0.0001 |
| Control | -2.98 | 1.000 |  | -3.74 | 1.000 |
| *R. maidis* on pre-inhabited host by virus-free *R. padi* | 8.28 | < 0.0001 |  | 5.06 | < 0.0001 |
| *R. maidis* on pre-inhabited host by virus-free *R. maidis* | 0.72 | 0.1863 |  | -2.61 | 0.8633 |
|  | ***Rhopalosiphum padi*** | | | | |
| *R. padi* on pre-inhabited host by viruliferous *R. padi* with BYDV-PAV | 41.95 | < 0.0001 |  | 38.50 | < 0.0001 |
| *R. padi* on pre-inhabited host by viruliferous *R. maidis* with BYDV-RMV | 12.35 | < 0.0001 |  | 16.51 | < 0.0001 |
| Control | -4.25 | 1.000 |  | -4.56 | 1.0000 |
| *R. padi* on pre-inhabited host by virus-free *R. maidis* | 13.62 | < 0.0001 |  | 0.17 | 0.0394 |
| *R. padi* on pre-inhabited host by virus-free *R. padi* | 1.81 | 0.0025 |  | -3.02 | 0.8158 |

**Table S3** Effects of pre-inhabitation of aphids and virus infection on plant nutritional quality, differences in carbohydrates, sterols, essential and non-essential amino acids of leaves previously inhabited by viruliferous aphids with BYDV-PAV or BYDV-RMV, pre-inhabited virus-free, and virus-free plants. Multivariate analysis of variance for nutritional factors (MANOVA Wilks’ Lambda=0.0022 , *F* _12,24.10_ =18.09, *P* ≤ 0.03), followed by univariate variance analysis (ANOVA) and Dunnet’s test.

| **Nutrient** | **Absolute difference LSD** | ***P*** |
| --- | --- | --- |
|  |  |  |
| **Carbohydrates** | | |
| Glucose (*F*_3,12_ =29.92, *P*< 0.0001 ) |  |  |
| Pre-inhabited + BYDV-PAV | 10.71 | <0.0001 |
| Pre-inhabited + BYDV-RMV | -5.70 | 0.9915 |
| Pre-inhabited | -5.21 | 0.9867 |
| Control | -5.70 | 1.0000 |
|  |  |  |
| Fructose (*F*_3,12_ =7.39 , *P*= 0.0046) |  |  |
| Pre-inhabited + BYDV-PAV | 4.05 | 0.0083 |
| Pre-inhabited + BYDV-RMV | -6.11 | 0.5279 |
| Pre-inhabited | -8.06 | 0.8310 |
| Control | -10.4 | 1.0000 |
|  |  |  |
| **Sterols** | | |
| Total sterols (Campesterol, stigmasterol, sitosterol) (*F*_3,12_ =4.92 , *P*= 0.187 ) | | |
| Pre-inhabited + BYDV-PAV | 1.89 | 0.0152 |
| Pre-inhabited + BYDV-RMV | -3.72 | 0.4046 |
| Pre-inhabited | -7.53 | 0.9999 |
| Control | -0.01 | 1.0000 |
|  |  |  |
|  |  |  |
| **Essential amino acids** | | |
| Arginine (*F*_3,12_= 3.43, *P*< 0.0001 ) |  |  |
| Pre-inhabited + BYDV-PAV | 0.01 | <0.0001 |
| Pre-inhabited + BYDV-RMV | -0.01 | 0.9257 |
| Pre-inhabited | -0.01 | 0.9966 |
| Control | -0.01 | 1.0000 |
|  |  |  |
| Leucine (*F*_3,12_ = 10.84, *P*= 0.0010) |  |  |
| Pre-inhabited + BYDV-PAV | 0.052 | 0.0009 |
| Pre-inhabited + BYDV-RMV | -0.02 | 0.3021 |
| Pre-inhabited | -0.06 | 1.000 |
| Control | -0.06 | 1.0000 |
|  |  |  |
| Methionine (*F*_3,12_ _=_27.34, *P*< 0.0001) |  |  |
| Pre-inhabited + BYDV-PAV | 0.01 | <0.0001 |
| Pre-inhabited + BYDV-RMV | -2e-3 | 0.2537 |
| Pre-inhabited | -2e-3 | 0.2746 |
| Control | -0.01 | 1.0000 |
|  |  |  |
| Phenylalanine (*F*_3,12_=8.85, *P*= 0.0023) | | |
| Pre-inhabited + BYDV-PAV | 0.005 | 0.0015 |
| Pre-inhabited + BYDV-RMV | -3e-3 | 0.3756 |
| Pre-inhabited | -0.01 | 0.9325 |
| Control | -0.01 | 1.0000 |
|  |  |  |
| Tryptophan ( *F*_3,12_ =421.92, *P*< 0.001) |  |  |
| Pre-inhabited + BYDV-PAV | 1.46 | <0.0001 |
| Pre-inhabited + BYDV-RMV | 0.5 | <0.001 |
| Pre-inhabited | -0.14 | 0.9986 |
| Control | -0.14 | 1.0000 |
|  |  |  |
| Valine (*F*_3,12_=989.73, *P*< 0.0001) |  |  |
| Pre-inhabited + BYDV-PAV | 1.17 | <0.0001 |
| Pre-inhabited + BYDV-RMV | 0.79 | <0.0001 |
| Pre-inhabited | -0.07 | 0.9959 |
| Control | -0.08 | 1.0000 |
|  |  |  |
|  |  |  |
| Isoleucine (*F*_3,12_= 715.25, *P*< 0.0001) |  |  |
| Pre-inhabited + BYDV-PAV | 1.21 | <0.0001 |
| Pre-inhabited + BYDV-RMV | -0.08 | 1.0000 |
| Pre-inhabited | -0.09 | 0.9813 |
| Control | -0.09 | 1.0000 |
|  |  |  |
| Lysine (*F*_3,12_=83.16, *P*< 0.0001) |  |  |
| Pre-inhabited + BYDV-PAV | 0.033 | <0.0001 |
| Pre-inhabited + BYDV-RMV | -0.01 | 0.6403 |
| Pre-inhabited | -0.01 | 0.9984 |
| Control | -0.01 | 1.0000 |
|  |  |  |
| Threonine (*F*_3,12_=339.72, *P*< 0.0001) |  |  |
| Pre-inhabited + BYDV-PAV | 1.28 | <0.0001 |
| Pre-inhabited + BYDV-RMV | -0.09 | 0.8503 |
| Pre-inhabited | -0.13 | 0.9908 |
| Control | -0.15 | 1.0000 |
|  |  |  |
|  |  |  |
| **Non-essential amino acids** |  |  |
| Alanine (*F*_3,12_ = 27.97, *P*< 0.0001) |  |  |
| Pre-inhabited + BYDV-PAV | .849 | 0.0001 |
| Pre-inhabited + BYDV-RMV | -0.39 | 0.9422 |
| Pre-inhabited | -0.45 | 0.9998 |
| Control | -0.46 | 1.0000 |
|  |  |  |
| Asparagine (*F*_3,12_*=* 41.94, *P*= 0.0004) |  |  |
| Pre-inhabited + BYDV-PAV | 0.015 | <0.0001 |
| Pre-inhabited + BYDV-RMV | -0.01 | 0.9729 |
| Pre-inhabited | -5e-3 | 0.8747 |
| Control | -0.01 | 1.0000 |
|  |  |  |
| Aspartic acid (*F*_3,12_= 18.95 , *P*= 0.0004) |  |  |
| Pre-inhabited + BYDV-PAV | 0.76 | <0.0001 |
| Pre-inhabited + BYDV-RMV | -0.37 | 0.7632 |
| Pre-inhabited | -0.47 | 0.9787 |
| Control | -0.53 | 1.0000 |
|  |  |  |
|  |  |  |
| Glutamic acid (*F*_3,12_*=* 30.28, *P*< 0.0001) | | |
| Pre-inhabited + BYDV-PAV | 1.59 | < 0.0001 |
| Pre-inhabited + BYDV-RMV | -0.05 | 0.0684 |
| Pre-inhabited | -0.76 | 0.9999 |
| Control | -0.77 | 1.0000 |
|  |  |  |
| Glutamine (*F*_3,12_= 12.16 , *P*= 0.0006) |  |  |
| Pre-inhabited + BYDV-PAV | 0.05 | 0.0017 |
| Pre-inhabited + BYDV-RMV | -0.05 | 0.7384 |
| Pre-inhabited | -0.08 | 1.0000 |
| Control | -0.08 | 1.0000 |
|  |  |  |
| Glycine (*F*_3,12=_47.70, *P*< 0.0001) |  |  |
| Pre-inhabited + BYDV-PAV | 0.01 | < 0.0001 |
| Pre-inhabited + BYDV-RMV | -5e-3 | 0.9214 |
| Pre-inhabited | -0.01 | 0.9994 |
| Control | -0.08 | 1.0000 |
|  |  |  |
| Serine (*F*_3,12_=283.99, *P*< 0.0001) |  |  |
| Pre-inhabited + BYDV-PAV | 1.19 | <0.0001 |
| Pre-inhabited + BYDV-RMV | -0.10 | 0.7079 |
| Pre-inhabited | -0.15 | 0.9998 |
| Control | -0.36 | 1.0000 |
|  |  |  |
| Tyrosine(*F*_3,12_=183.36, *P*< 0.0001) |  |  |
| Pre-inhabited + BYDV-PAV | 1.21 | <0.0001 |
| Pre-inhabited + BYDV-RMV | -0.19 | 0.9994 |
| Pre-inhabited | -0.20 | 1.0000 |
| Control | -0.20 | 1.0000 |
